# Maternity service organisational interventions that aim to reduce caesarean section: a systematic review and meta-analyses

**DOI:** 10.1186/s12884-019-2351-2

**Published:** 2019-07-09

**Authors:** Anna Chapman, Cate Nagle, Debra Bick, Rebecca Lindberg, Bridie Kent, Justin Calache, Alison M. Hutchinson

**Affiliations:** 1 School of Nursing and Midwifery, Centre for Quality and Patient Safety Research, Deakin University, Geelong, VIC Australia; 2 Monash Medical Centre, Monash Health, Level 2 I Block, 246 Clayton Rd, Clayton, 3168 VIC Australia; 3 Centre for Nursing and Midwifery Research, James Cook University, Townsville, Queensland Australia; 4 Townsville Hospital and Health Service, Townsville, Queensland Australia; 5 Warwick Clinical Trials Unit, Warwick Medical School, University of Warwick, Coventry, UK; 6 Institute for Physical Activity and Nutrition, School of Exercise and Nutrition Sciences, Deakin University, Geelong, Victoria Australia; 7 Faculty of Health and Human Sciences, University of Plymouth, Plymouth, Devon UK

**Keywords:** Caesarean section, Systematic review, Meta-analysis, Organisational interventions, Childbirth, Maternity service, Midwife-led care

## Abstract

**Background:**

Caesarean sections (CSs) are associated with increased maternal and perinatal morbidity, yet rates continue to increase within most countries. Effective interventions are required to reduce the number of non-medically indicated CSs and improve outcomes for women and infants. This paper reports findings of a systematic review of literature related to maternity service organisational interventions that have a primary intention of improving CS rates.

**Method:**

A three-phase search strategy was implemented to identify studies utilising organisational interventions to improve CS rates in maternity services. The database search (including Cochrane CENTRAL, CINAHL, MEDLINE, Maternity and Infant Care, EMBASE and SCOPUS) was restricted to peer-reviewed journal articles published from 1 January 1980 to 31 December 2017. Reference lists of relevant reviews and included studies were also searched. Primary outcomes were overall, planned, and unplanned CS rates. Secondary outcomes included a suite of birth outcomes. A series of meta-analyses were performed in RevMan, separated by type of organisational intervention and outcome of interest. Summary risk ratios with 95% confidence intervals were presented as the effect measure. Effect sizes were pooled using a random-effects model.

**Results:**

Fifteen articles were included in the systematic review, nine of which were included in at least one meta-analysis. Results indicated that, compared with women allocated to usual care, women allocated to midwife-led models of care implemented across pregnancy, labour and birth, and the postnatal period were, on average, less likely to experience CS (overall) (average RR 0.83, 95% CI 0.73 to 0.96), planned CS (average RR 0.75, 95% CI 0.61 to 0.93), and episiotomy (average RR 0.84, 95% CI 0.74 to 0.95). Narratively, audit and feedback, and a hospital policy of mandatory second opinion for CS, were identified as interventions that have potential to reduce CS rates.

**Conclusion:**

Maternity service leaders should consider the adoption of midwife-led models of care across the maternity episode within their organisations, particularly for women classified as low-risk. Additional studies are required that utilise either audit and feedback, or a hospital policy of mandatory second opinion for CS, to facilitate the quantification of intervention effects within future reviews.

**PROSPERO registration:**

CRD42016039458; prospectively registered.

**Electronic supplementary material:**

The online version of this article (10.1186/s12884-019-2351-2) contains supplementary material, which is available to authorized users.

## Background

Caesarean section (CS), when medically indicated, can effectively prevent maternal and perinatal morbidity and mortality [1]. However, steady increases in CS rates within the majority of regions worldwide have generated concern about the utilisation of this procedure when not medically justified [2]. The average CS growth rate among Organisation for Economic Cooperation and Development (OECD) countries increased from 20% in 2000 to 28.6% in 2016, with the highest rates observed in Turkey, Chile and Mexico (≥46%) [3]. Recent epidemiological research has indicated that population-level CS rates above 19% are not associated with reductions in maternal and neonatal mortality [1, 4]. Consequently, appropriate utilisation of CS is a central focus for health professionals and health systems globally, with equitable access and patient safety considered paramount.

Global concern for patient safety in response to rising rates of CS is warranted. While CS effectively expedites birth in obstetric emergencies, CSs when not medically indicated, are associated with an increased risk of short- and long-term complications for women and their infants. For women, a CS can increase the risk of bladder injuries, postpartum infections, anaesthetic complications, obstetric shock, hysterectomy, thromboembolism and psychological distress [5]. In contrast, benefits of planned vaginal birth, compared with planned CS birth among low-risk women, include lower rates of infection and faster recovery [6]. For the term infant, medically unjustified CSs have been associated with increased risk of neonatal intensive care admission and respiratory problems, and a reduced likelihood of breastfeeding initiation [7, 8]. The economic burden of CSs is also worth noting. Compared with vaginal delivery, higher mean costs have been associated with CS in low-risk populations [9].

A multitude of reasons are linked to the increasing trend in CS rates particularly within high-resource settings, including a shift in maternal socio-demographic characteristics, scheduling convenience, changes to professional practice styles, an increase in pre-existing maternal medical conditions (i.e. diabetes, obesity), malpractice liability concerns, and an enhanced maternal preference for the procedure [10]. These factors have been targeted in a variety of interventions designed to safely reduce CS rates in high-use settings. To date, the effectiveness of interventions directed at both women and healthcare providers is limited, with very few studies displaying clinically meaningful effects [11]. Organisational interventions, on the other hand, have recently been posited as having the most potential for reducing the rising trend in CS rates [12]. As outlined by Cochrane Effective Practice and Organisation of Care Group, an organisational intervention is one which*“…involves a change in the structure or delivery of health care … a change in who delivers health care, how care is organised, or where care is delivered*…” [13]. Not since 2007 has a systematic review of maternity service organisational interventions been performed; in this review, positive effects on CS rates were observed for audit and feedback, quality improvement, and multifaceted strategies (e.g. combination of education, audit and feedback and implementation of practice guidelines) [14]. Additionally, two recent Cochrane reviews have examined the effects of midwife-led models of care [15] and one-to-one support in labour [16] on CS, with positive effects observed for one-to-one labour support only. These reviews did not specifically limit inclusion to interventions with a primary outcome of CS; furthermore, the review of one-to-one support in labour included studies that utilised informal support persons within interventions. It is therefore timely that a systematic review of organisational interventions specifically designed to improve CS rates in maternity services be performed.

This systematic review and meta-analyses was designed to synthesise literature related to maternity service organisational interventions that had a primary aim of improving CS rates. Additionally, this work sought to quantify the effectiveness of relevant organisational interventions on CS rates, relative to comparator conditions. Findings of this review have potential to inform future policies and programs designed to optimise the utilisation of CS within maternity service settings.

## Methods

This systematic review complies with the Preferred Reporting Items for Systematic reviews and Meta-Analyses (PRISMA) statement [17]. The protocol for the review was prospectively registered in PROSPERO (CRD42016039458), and the study protocol, describing the rationale and methods in detail, has been published [18].

### Data sources and search strategy

A three-phase search strategy was implemented to identify literature relevant to the current investigation. Firstly, electronic databases were searched, structured according to the nuances for each database (the Cochrane Central Register of Controlled Trials, CINAHL, MEDLINE, Maternity and Infant Care, EMBASE and SCOPUS) using a combination of keywords relating to CS and maternity service organisational interventions and controlled vocabulary. The search was restricted to peer-reviewed, journal articles published from 1 January 1980 to 31 December 2017. No restrictions on language or setting were applied. An example of the MEDLINE search strategy is included as a Additional file 1. Secondly, reference lists of relevant reviews captured within the database search were manually cross-checked to identify additional articles. Thirdly, reference lists of all included studies were reviewed to identify additional eligible references. Two reviewers independently assessed titles and abstracts of studies for inclusion at all three stages. Selections were then compared and where discrepancies occurred, consensus was reached through discussion with a third reviewer.

### Eligibility criteria

Studies were only included if they reported on maternity service organisational interventions designed to reduce CS rates (including planned and unplanned CS). Eligible study designs were those classified as randomised controlled trials (RCTs), cluster-RCTs, quasi-RCTs, controlled before and after studies, and interrupted time series studies. Inclusion criteria were based on the following PICO (participants, interventions, comparators, outcomes) criteria:

- *Participants and settings:* Maternity care clinicians, including midwives, obstetricians, nurses, paediatricians, family doctors and anaesthetists, maternity care managers and maternity care educators. Eligible settings were obstetric-led maternity services able to provide support for women undergoing planned or unplanned caesarean birth.
- *Interventions:* Eligible maternity service organisational interventions included models of care, audit and feedback, hospital policy/protocol interventions, labour assessment triage, incentives, education, and reminder mechanisms. Intervention strategies aiming to reduce CS rates were permitted to be either single component, or multi-faceted (i.e. complex interventions).
- *Comparators:* An appropriate comparator group was specified as no intervention, or usual/routine care.
- *Outcomes:* Studies that reported objectively measured or self-reported (using validated instruments) outcomes. Planned, unplanned and overall CS rates were the primary outcome measures. Studies that did not specify CS rates as the primary outcome measure were not eligible for inclusion. Where studies did not explicitly differentiate between primary and secondary outcome measures, studies were included if CS rate was deemed by the systematic review authors as an outcome of priority. Secondary outcomes of interest included labour interventions (e.g. epidural use, labour augmentation), maternal adverse events (e.g. postpartum haemorrhage, third or fourth degree perineal tear), neonatal outcomes (e.g. admission to neonatal intensive care unit, Apgar scores), breastfeeding initiation, maternal/newborn duration of inpatient stay, maternal experiences of care, adherence to best practice guidelines by health professionals, health professionals’ satisfaction, confidence, competence, attitudes, knowledge and self-efficacy.

Medical interventions (e.g. induction of labour, episiotomy, instrumental vaginal delivery), lifestyle interventions (e.g. nutrition and physical activity programs for pregnant women), labour interventions (e.g. water births, epidural analgesia, augmentation of labour), and interventions utilising active management of labour, were not deemed eligible maternity service organisational interventions and were excluded. Additionally, doulas (also referred to as birth companions) and informal support persons were not classified as eligible health care providers in this review; hence, any studies targeting these providers were also excluded.

### Data extraction

Two reviewers independently extracted data from included studies using a standardised template. The resultant data included study and sample characteristics (e.g. research aim, setting, sample size), design features (e.g. intervention type/s and regimen of intervention condition/s) and study results (e.g. RR, 95% CI). Only data reported in the original papers were used for extraction, and no attempts were made to contact corresponding authors to obtain unpublished data.

### Risk of bias/quality appraisal

The Cochrane Collaboration’s tool for assessing risk of bias was used for all included studies that utilised an RCT or cluster RCT design [19]. This tool examines randomisation procedure and allocation concealment (selection bias); blinding of participants and personnel (performance bias); blinding of outcome assessors (detection bias); incomplete outcome data (attrition bias); selective outcome reporting (reporting bias); and other sources of bias (e.g. baseline imbalance, recruitment issues etc.). A positive classification indicated a low risk of bias, while a negative classification indicated a high risk of bias. An unclear classification was given when there was insufficient information within manuscripts to adequately assess risk of bias.

The Quality Assessment Tool for Quantitative Studies [20] was used for all other research designs. This tool addresses eight domains: selection bias; study design; confounders; blinding; data collection; participant withdrawals; intervention integrity and analysis. The quality assessment across the eight domains allows an overall quality rating to be determined for each study: ‘strong’, ‘moderate’ or ‘weak’.

Two reviewers independently performed all risk of bias/quality appraisal assessments, with consensus reached through joint discussion.

### Data synthesis and analysis

For studies utilising an RCT design, a series of meta-analyses were performed that were separated by type of maternity service organisational intervention and outcome of interest. A minimum of three studies per outcome was considered adequate for a meta-analysis. Where a meta-analysis was not possible (e.g. < 3 studies per outcome per intervention type), the results were synthesised and discussed narratively.

Quantitative data from included studies were analysed using RevMan 5.3 software [21]. Secondary outcome variables reported within included studies were mapped to assist with the selection and prioritisation of secondary outcomes to be reported in this review. Secondary outcomes were included in this review when a minimum of three included studies reported on a given outcome. All meta-analyses performed involved the use of dichotomous data, and as such, summary risk ratio with 95% confidence intervals were presented as the effect measure. Effects were pooled using a random-effects model (Mantel-Haenszel method). In contrast to a fixed-effects model that assumes all included studies share one true effect, a random-effects model assumes each study estimates a different underlying true effect, and produces a summary effect that is an estimate of the mean of a distribution of true effects [22]. After obtaining the full set of included studies, and noting the substantial clinical and methodological heterogeneity between studies (i.e. differences in intervention regimens, country of origin), a random-effects model was selected in preference to a fixed-effects model, as it was deemed unreasonable to assume that all included studies shared a common true effect.

Heterogeneity in each meta-analysis was examined using the I^2^, Tau^2^, and χ^2^ statistic with associated significance value. A *p*-value for the χ^2^ statistic of 0.10 (rather than 0.05) was used to indicate statistically significant heterogeneity due to the limitations of this test when there are low numbers of included studies [23, 24]. The following classifications for the I^2^ statistic were used to determine the degree of heterogeneity: not important (0–40%), moderate heterogeneity (30–60%), substantial heterogeneity (50–90%) and considerable heterogeneity (75–100%) [23]. Tau^2^ values were examined and subsequently reported within the text when heterogeneity was found to be significant, or the I^2^ statistic was ≥30%.

Subgroup analyses to examine potential sources of heterogeneity were pre-specified that utilised the Robson classification system [25] to differentiate between interventions by type of maternal group [18]. This planned assessment was not possible however, as included studies utilised maternal participant groups that were either not described in sufficient detail or encompassed a diverse mix of maternal groups (not separated in analysis). Similarly, an assessment of publication bias/small study effects could not be performed due to the limited number of included studies (< 10 studies).

It was initially planned to combine results from cluster RCTs with individually-randomised RCTs if there was minimal clinical and methodological heterogeneity between the studies, and the interaction between the intervention effect and choice of randomisation units were deemed unlikely [18]. However, only RCTs with a parallel design were ultimately eligible for inclusion within the quantitative synthesis. Had any cluster RCTs been eligible for a least one meta-analysis, the standard error of the cluster RCT would have been adjusted using the reported intracluster correlation coefficient. Heterogeneity in the unit of randomisation would also have been acknowledged, and a sensitivity analysis to investigate the effects of the randomisation unit would have been performed.

## Results

**Fig. 1:**
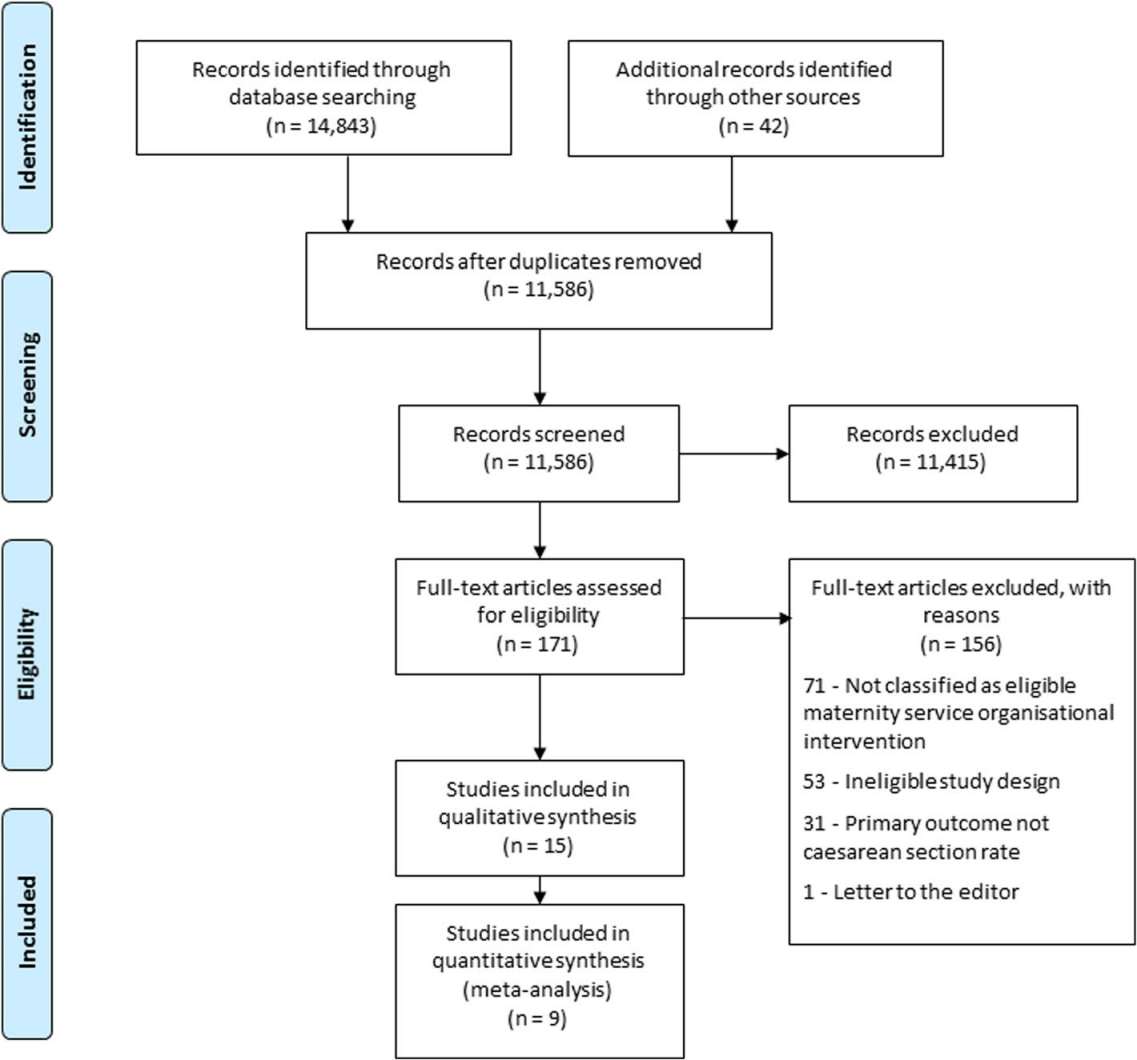
PRISMA flow diagram

The three-phase search strategy yielded 11,586 citations after duplicates were removed. The first phase of screening identified 171 abstracts as potentially eligible; full texts were subsequently retrieved and assessed for inclusion. One hundred and fifty-six articles were excluded in the second phase of screening as they did not meet specified criteria. In total, 15 articles were included in the systematic review, nine of which were appropriate for inclusion in at least one meta-analysis. A PRISMA flow diagram details the results of the systematic search and screening process (Fig. 1).

The characteristics of included studies are provided in Table 1. The majority of studies utilised a parallel RCT design (k = 12, 80%), with the remaining studies adopting either a cluster RCT design (k = 2, 13%) [26, 27], or quasi-experimental controlled design (k = 1, 7%) [28]. Year of publication ranged from 1992 to 2015, with the majority of studies published from 2001 (k = 11, 73%). All studies were published in English, and all interventions were implemented in hospitals; however, countries varied: USA/Canada (k = 6, 40%), Australia (k = 4, 27%), Iran (k = 2, 13%), Ireland (k = 1, 6.7%), China (k = 1, 6.7%), and Latin America (k = 1, 6.7%). Birth outcome data were largely obtained via medical record audits for samples of women, with samples ranging in size from 100 to 149,276 participants (mean 14,665 ± 39,509; median 1172). Midwife-led models of care were the most commonly utilised maternity service organisational intervention (k = 8, 53%), which were primarily implemented across pregnancy, labour, birth, and the postnatal period (postnatal periods ranged from early through to 6-weeks), (k = 6). In the six studies where the midwife-led model of care traversed all maternal periods, two studies utilised a caseload approach [29, 30], with the other four studies using a team-based approach [31–34]. Other maternity service organisational interventions utilised included continuous midwifery care (also referred to as one-to-one/continuous labour support) (k = 3, 20%) [35–37], audit and feedback to promote implementation of evidence-based practice (k = 1, 6.7%) [27], labour assessment triage (at home vs hospital) (k = 1, 6.7%) [38], hospital policy of mandatory second opinion for CS (k = 1, 6.7%) [26], and hospital protocols for management of pregnancy complications (k = 1, 6.7%) [28]. In the majority of studies, these additional types of organisational intervention were implemented during labour and birth (k = 5), while in one study the intervention was implemented only during pregnancy [28].

**Table 1:** Characteristics of included studies

First author [year]	Research aim	Study design	Country/Setting (site numbers)	Maternal group	Sample size	Intervention type/ Maternal period of study	Intervention and comparison regimen	Results for primary outcome
Althabe [2004]	To test the hypothesis that a hospital policy of mandatory second opinion reduces hospital CS rate by 25% without increasing maternal and perinatal morbidity and mortality.	Cluster RCT	Argentina, Brazil, Cuba, Guatemala, Mexico/Hospital (36 sites)	Pregnant women giving birth	36 hospitals randomised/34 hospitals (17 in each arm) and 149,276 women (I: 70410/C: 78866) at completion.	Hospital policy of mandatory second opinion/Labour and Birth	*Intervention group:* 6-month implementation of a hospital policy of mandatory second opinion before non-emergency CS. Second opinion was sought by the attending physician with a consultant physician with clinical qualifications equal to or higher than the attending physician. Cases were discussed in relation to a suite of prepared guidelines. *Comparison group:* Routine care.	+ (relative rate reduction 7.3%, *p* = 0.044)
Begley [2011]	To compare midwife-led versus consultant-led care for healthy, pregnant women without risk factors for labour and delivery.	Pragmatic RCT	Ireland/Hospital (2 sites)	Pregnant women (17–39 years, healthy, < 24 weeks gestation at booking, low-risk)	1653 women (I: 1101/C: 552)	Midwife-led model of care/Pregnancy, Labour and Birth, Postnatal	*Midwife-led care group:* Care provided by the same small group of midwives in the midwifery-led unit consisting of pregnancy care (including assessment) by midwives and, if desired, the woman’s GP; labour and birth care by midwives; postnatal care by midwives for up to two days. On discharge, midwives visited women at home, and/or provided telephone support, up to the 7th postpartum day, when care was transferred to the Public Health Nursing service. *Comparison group:* Standard care consisting of pregnancy care provided by obstetricians and, if desired, the woman’s GP, supported by the hospital medical team with assistance from midwives; labour and birth care by midwives unless complications developed, with consultant overview; and postpartum care by midwives, overseen by consultants; discharged into the care of Public Health Nurses.	X (RR 0.97, 95% CI 0.76 to 1.24)
Chaillet [2015]	To assess whether a multifaceted intervention to promote professional onsite training with audit and feedback would reduce the rate of caesarean delivery and other maternal and neonatal outcomes.	Cluster RCT	Canada/Hospital (32 sites)	Pregnant women giving birth	32 hospitals (16 in each arm). Primary analysis based on 105,351 women (pre-intervention 53,086; post-intervention 52,265)	Audit and feedback, Implementation of evidence-based practice/Labour and Birth	*Intervention group*: Multifaceted 1.5-year intervention, consisting of training in evidence-based clinical practices, audits of indications for caesarean delivery, the provision of informal and formal feedback to health professionals, and implementation of best practices. *Comparison group:* No intervention.	+ (Adj. OR 0.90, 95% CI 0.80 to 0.99, *p* = 0.04)
Chambliss [1992]	To test the hypothesis that the low caesarean birth rate on the midwifery service was the result of patient selection bias.	RCT	USA/Hospital (1 site)	Pregnant women (16–45 years, singleton vertex presentation, 36–42 weeks gestation, foetal size estimation of 2500-4000 g) in labour	487 women (I: 234/C: 253)	Midwife-led model of care/Labour and Birth	*Intervention group*: Women were managed in the birth centre using previously established protocols. Care exclusively provided by midwives, unless physician consultation was sought. Midwife-led careliberally uses ambulation, varied positions for birth, and a support person as an integral part of labour management. *Comparison group:* Women were managed in the labour and birthing ward. This ward is directed by a senior resident primarily responsible for management decisions, in consultation with an attending physician. The service rarely uses ambulation, does not use birthing beds (instead a lithotomy position on a delivery table), patients rarely have a coach or support person, and epidural anaesthesia is common.	X (*p* > 0.05)
Gagnon [1997]	To compare the risks and benefits of one-to-one nurse labour support with usual labour and birth care.	RCT	Canada/Hospital (1 site)	Pregnant women (nulliparous, ≥37 weeks gestation, singleton) in labour	413 women (I: 209/C: 204)	Continuous Midwifery Care/Labour and Birth	*Intervention group:* Continuous one-to-one nursing care from the time of randomisation until one hour after birth. During this time, in addition to the usual labour and birth care (including foetal monitoring and intravenous regulation), the nurse provided physical comfort, emotional support, and instruction on relaxation and coping techniques to the woman; gave support to the expectant father; contacted the attending physician; contacted the anaesthesiologist when appropriate; and updated the unit staff on the progress of labour. *Comparison group:* Usual labour and birth care.	X (RR 0.86, 95% CI 0.54 to 1.36)
Gu [2013]	To develop and implement a midwife-led pregnancy clinic service in China and explore its effect on childbirth outcomes, psychological state and satisfaction.	RCT	China/ Hospital (1 site)	Pregnant women (Mandarin-speaking, primiparous 29–30 weeks gestation at recruitment; low risk, singleton)	110 women randomised (I: 55/ C:55)/ 106 women included in final analysis (I: 53/C:53)	Midwife-led model of care/ Pregnancy ± Labour and Birth/ Postnatal (up to 2 h)	*Intervention group:* Women received individual care from a specially trained, experienced midwife following each pregnancy obstetrician appointment. The midwife typically focussed on pregnancy check-ups, consultation, making birth plans, parent education, and collaborated with obstetricians. The midwife was on call for the woman’s labour and birth except in designated circumstances, in which case an associate midwife would be present. Each women had a chance of chance of having continuous one-to-one care from the onset of labour to 2 h postpartum. *Comparison group:* Women received routine obstetrician-led pregnancy care. This included consultations with obstetricians who could differ at each visit; being cared for in labour and birth by rostered midwives and obstetricians. Each woman had a chance of receiving one-to-one continuity of care by a duty midwife from the onset of labour to 2 h postpartum.	+ (Difference − 22.64, 95% CI −41.60 to −3.69, *p* = 0.019)
Harvey [1996]	To determine if nurse-midwifery care was as effective as traditional medical care for low-risk women with respect to clinical outcomes.	RCT	Canada/ Hospital (1 site)	Pregnant women (low-risk, ≥20 weeks gestation at study entry)	194 women (I: 101/ C: 93)	Midwife-led model of care/ Pregnancy, Labour and Birth, Postnatal	*Intervention group:* Women received care from a team of seven nurse-midwives who provided complete management of those with uncomplicated pregnancies. Protocols and guidelines for the care were based on the midwifery philosophy and standards of practice developed by the Alberta Association of Midwives. Women were seen during pregnancy in the nurse-midwifery clinic. A nurse-midwifery team member provided care throughout the labour, birth, and immediate postpartum period. A member of the team carried out postpartum follow-up in the postpartum unit or at home, and a 6-week follow-up visit was performed in the midwifery clinic. *Comparison group:* Women selected their physician through standard referral processes, and were free to use any family practice physician or obstetrician in the area.	+ (*p* = 0.01, 95% CI for difference 2.9 to 19.3%)
Hodnett [2002]	To evaluate the effectiveness of nurses as providers of labour support in hospitals.	RCT	USA & Canada/ Hospital (13 sites)	Pregnant women (singleton or twin, ≥34 weeks gestation) in established labour	6915 women (I: 3454/ C: 3461)	Continuous Midwifery Care/Labour and Birth	*Intervention group:* Women received continuous labour support (minimum 80% nurse time from randomisation to birth) from specially trained nurses. *Comparison group:* Usual care.	X (*p* = 0.44)
Homer [2001]	To test whether a new community-based model of continuity of care provided by midwives and obstetricians improved maternal clinical outcomes, in particular a reduced CS rate.	RCT	Australia/ Hospital (1 site)	Pregnant women (< 24 weeks gestation at first visit, < 2 prior caesarean deliveries)	1089 women (I: 550/ C: 539)	Midwife-led model of care/Pregnancy, Labour and Birth, Postnatal	*Intervention group:* Community-based model with a team of six full-time midwives - the emphasis was on continuity of care (a consistent team approach) rather than carer (the same midwife). Two midwives and an obstetrician or obstetric registrar attended each clinic. Two teams were involved and one midwife from each was always on call for women in labour and to provide advice and information. After the birth, women could either choose to remain in hospital for postnatal care with community-based midwives or be discharged early and receive domiciliary care by the community-based midwives. *Comparison group:* Standard care provided in the hospital-based pregnancy clinic, the birthing suite and the postnatal ward. Midwives and doctors saw women in the pregnancy clinic. Women with risks were seen by an obstetrician or obstetric registrar; low-risk women by midwives. Hospital-based pregnancy care included visits to the women’s GP (i.e. shared care). Midwives and doctors on duty provided care in the birthing suite and the postnatal ward. Standard care was characterised by a lack of continuity of care across the pregnancy, labour and birth and postnatal periods as a large number of clinicians provided care.	+ (OR 0.6, 95% CI 0.4 to 0.9, *p* = 0.02)
Janssen [2006]	To compare rates of caesarean birth among women who were triaged by obstetric nurses at home visits vs telephone.	RCT	Canada/ Hospital & Home (7 sites)	Pregnant women (16–42 years, 37–41 weeks gestation, nulliparous, singleton vertex presentation, ± induced on an outpatient basis with prostaglandins) in labour	1459 women (I: 728/ C: 731)	Labour Assessment Triage/ Labour	*Home-triage (intervention) group:* Women received a nursing assessment at home (identical to that received by the control group over telephone) and received an assessment that included maternal vital signs, abdominal palpation, auscultation of the foetal heart rate, assessment of contractions, and examination of the cervix. Comfort measures were taught to the woman and her support person/s as needed. The study nurse contacted the primary physician by telephone following assessment and a joint decision made whether to remain at home longer. If needed, a woman could be assessed at home more than once. Women could access the study nurse via phone at any time. *Telephone-triage (comparison) group:* Women were asked questions over the telephone about the nature of contractions, presence of bloody show, status of membranes, colour of amniotic fluid, presence of bleeding, nature of foetal movements, and their own coping assessment. Suggestions for coping were made over the phone. Women could access the study nurse via phone at any time.	X (RR: 1.12, 95% CI 0.94 to 1.32)
Kashanian [2010]	To evaluate the effect of continuous support provided by midwives during labour on the duration of the different stages of labour and the rate of caesarean delivery.	RCT	Iran/ Hospital (1 site)	Pregnant women (nulliparous, 18–34 years, low-risk, 38–42 weeks gestation, singleton cephalic presentation, estimated foetal weight of 2500–3400 g, cervical dilatation of 3–4 cm with appropriate contractions) in labour	100 women (I: 50/ C: 50)	Continuous Midwifery Care/ Labour and Birth	*Intervention group:* Women were shown to an isolated room and were supported by an experienced midwife. Women were free to choose their position, and were able to eat and walk about freely. During labour, the midwife explained the process of labour and the importance of body relaxation. Midwife-led support included close physical proximity, touch, eye contact with the labouring women, and teaching, reassurance, and encouragement. The midwife remained with the woman throughout labour and birth, and applied warm or cold packs to the woman’s back, abdomen, or other parts of the body, as well as performing massage according to each woman’s request. *Comparison group:* Women were admitted to the labour ward, did not receive continuous support, and care followed the routine procedures. Women did not have a private room, did not receive one-to-one care, were not permitted food, and did not receive education and explanation about the labour process. The only persons allowed in the birthing room were nurses, midwives, and doctors.	+ (*p* = 0.026)
McLachlan [2012]	To determine whether primary midwife care (caseload midwifery) decreases the CS rate compared with standard maternity care.	RCT	Australia/ Hospital (1 site)	Pregnant women (< 24 completed weeks gestation, singleton pregnancy, low obstetric risk at recruitment)	2314 women randomised (I: 1156/ C: 1158)/ 2286 included in final analysis (I: 1142/ C: 1144)	Midwife-led model of care/ Pregnancy, Labour and Birth, Postnatal	*Intervention group:* Women received the majority of their care from a ‘primary’ caseload midwife at the hospital. If complications developed, the primary midwife collaborated with obstetrician/ health professionals and continued to provide caseload care. The primary midwife was on call for the woman’s labour. Labour and birth care was provided in the hospital birthing suite. The primary midwife attended the hospital on most days to provide some postnatal care and provided domiciliary care following discharge. *Comparison group:* Standard care options for women included midwifery-led care with varying levels of continuity, obstetric trainee care and community-based care ‘shared’ between a GP and the participating hospital, where the GP provided the majority of pregnancy care. Women were cared for by rostered midwives and doctors for labour, birth and postnatal care.	+ (RR 0.78, 95% CI 0.67 to 0.91, *p* = 0.001)
Rowley [1995]	To compare continuity of care from a midwife team with routine care from a variety of doctors and midwives.	Stratified RCT	Australia/ Hospital (1 site)	Pregnant women	814 women (I: 405/ C: 409)	Midwife-led model of care/ Pregnancy, Labour and Birth, Postnatal	*Intervention group:* Women received pregnancy care, one-to-one labour and birth care, and early postnatal care by a team of 6 experienced and newly graduated midwives. Low-risk women were seen by a midwife at each visit but also had 3 consultations with a doctor. High-risk women had an individualised care plan devised in consultation with a doctor. They were seen by a midwife and a doctor at each visit, at a frequency determined by their risk status. Throughout labour, one of the team midwives provided care. *Comparison group:* Women received pregnancy care, labour and birth care, and early postnatal care by a variety of doctors and midwives working in the pregnancy clinic, birthing suite and postnatal area.	X (Planned CS: OR 0.82, 95% CI 0.45 to 1.52; Unplanned CS: 0.99, 95% CI 0.58 to 1.67)
Tracy [2013]	To assess the clinical and cost outcomes of caseload midwifery care for women irrespective of risk factors.	RCT	Australia/ Hospital (2 sites)	Pregnant women (≥18 years, < 24 weeks gestation at first visit, single foetus, planning to have vaginal birth)	1748 women (I: 871/ C: 877)	Midwife-led model of care/ Pregnancy, Labour and Birth, Postnatal	*Intervention group:* Women received pregnancy, labour and birth, and postnatal care in hospital and in the community from a named (or primary) caseload midwife, who worked within a small group known as a midwifery group practice. Women also received postnatal care at home from their caseload midwife for up to 6 weeks. *Comparison group:* Women chose from the standard hospital options for maternity care, which did not include substantial continuity of a midwifery carer. Standard hospital care was provided through antenatal clinics, labour wards, and postnatal wards, with care provided by rostered doctors and midwives.	X (OR 0·88, 95% CI 0·70 to 1·10, *p* = 0·26)
Yavangi [2013]	To evaluate the effectiveness of Iranian Ministry of Health and Medical Education protocols on CS rate trends.	Non-concurrent controlled quasi-experimental study	Iran/ Hospital (2 sites)	Pregnant women hospitalised with complications (premature rupture of membranes, prolonged pregnancy, pre-eclampsia, intrauterine growth retardation, vaginal bleeding, and premature labour in 1st /2nd trimester).	1172 women (I: 578/ C: 594)	Hospital protocols for pregnancy complications/ Pregnancy	*Intervention group:* Women hospitalised from December 2008 to April 2009 underwent interventions based on newly developed protocols for managing pregnancy complications. *Comparison group:* Women hospitalised from April 2008 to October 2008 were treated based on previous routine approaches and underwent no intervention based on the new protocols.	-(*p* = 0.001)

Note: “+” positive statistically significant finding that favoured the intervention group; “−” negative statistically significant finding that favoured the comparison group; “X” no statistically significant finding was obtained. Abbreviations: I (Intervention); C (Comparison); RCT (Randomised Controlled Trial); GP (General Practitioner); OR (Odds Ratio); RR (Risk Ratio); CI (Confidence Interval); CS (Caesarean Section)

Results of this systematic review are grouped and reported according to the type of maternity service organisational intervention. The presentation of results for primary outcomes have been prioritised, followed by secondary outcomes. The data for meta-analyses are presented first, followed by the narrative presentation of results. The estimated effects and heterogeneity of meta-analyses are summarised in Table 2. With regard to the narrative synthesis of outcomes, a small portion of secondary outcomes (maternal/newborn duration of inpatient stay, maternal experiences of care, adherence to best practice guidelines by health professionals, and health professionals’ satisfaction, confidence, competence, attitudes, knowledge and self-efficacy) were not reported in enough studies (< 3 of all included studies) to warrant inclusion.

**Table 2:** Summary of meta-analyses

Outcomes	K	N	Effect estimate			Heterogeneity			
			RR	95% CI	Z (p)	χ^2^	p	*I* ^2^	Tau^2^
Midwife-Led Care vs. Comparator									
Overall Caesarean Section	6	7784	0.83	0.73 to 0.96	2.63 (0.008)	8.32	0.14	40%	0.01
Planned Caesarean Section	4	5937	0.75	0.61 to 0.93	2.66 (0.008)	1.19	0.76	0%	0.00
Unplanned Caesarean Section	4	5937	0.87	0.73 to 1.03	1.65 (0.10)	4.55	0.21	34%	0.01
Induction of Labour	5	5498	0.91	0.79 to 1.04	1.43 (0.15)	6.06	0.19	34%	0.01
Epidural	6	7601	0.89	0.79 to 1.00	1.96 (0.05)	10.82	0.06	54%	0.01
Labour Augmentation	5	5498	0.97	0.73 to 1.29	0.23 (0.81)	48.99	< 0.00001	92%	0.09
Instrumental Vaginal Delivery	4	6776	0.96	0.86 to 1.07	0.78 (0.44)	1.82	0.61	0%	0.00
Episiotomy	6	6816	0.84	0.74 to 0.95	2.87 (0.004)	6.19	0.29	19%	0.00
Admission during Pregnancy	4	5304	0.94	0.80 to 1.11	0.77 (0.44)	7.06	0.07	58%	0.02
Apgar scores (< 7 at 5 min)	3	4711	0.94	0.66 to 1.33	0.37 (0.71)	0.88	0.64	0%	0.00
Admission to SCU/NICU	5	6599	0.80	0.62 to 1.04	1.66 (0.10)	8.54	0.07	53%	0.04
Continuous Midwifery Care vs. Comparator									
Overall Caesarean Section	3	7428	0.85	0.59 to 1.23	0.88 (0.38)	4.27	0.12	53%	0.06

Abbreviations: *K* number of studies, *N* number of participants, *RR* Risk Ratio, *CI* Confidence Interval, *Z* test for overall effect

### Midwife-led models of care

In the present review, a midwife-led model of care was defined as care where *“the midwife is the lead professional in the planning, organisation and delivery of care given to a woman”* [15]. A midwife-led model of care was implemented within eight included studies [29–34, 39, 40]. Six of these studies [29–34] were similar in terms of intervention regimen (midwife-led vs usual care), maternal period of study (pregnancy, labour and birth, and postnatal), and study design (RCT), and as such, results of these studies were pooled in a series of separate meta-analyses.

#### Overall caesarean sections

**Fig. 2:**
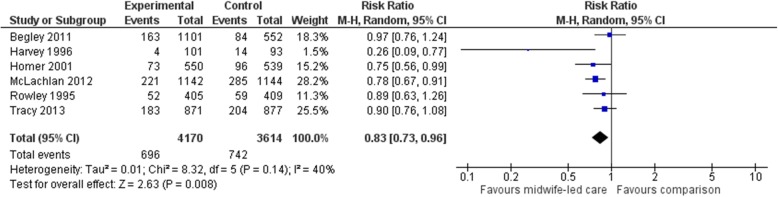
Forest plot for the outcome ‘overall caesarean section’ in the selected RCTs, comparing midwife-led models of care (implemented across pregnancy, labour and birth, and the postnatal period) with standard care

Six studies (*n* = 7784 participants) were included in a meta-analysis comparing midwife-led models of care (delivered over pregnancy, labour and birth, and postnatal periods) with a comparator group for overall CSs. Women allocated to midwife-led care were, on average, less likely to have a CS (average RR 0.83, 95% CI 0.73 to 0.96) (Fig. 2). The χ2 test for heterogeneity was not significant, with heterogeneity considered not important to moderate (χ2 = 8.32, *p* = 0.14, I^2^ = 40%, Tau^2^ = 0.01). Narratively, midwife-led models of care were utilised within two additional studies that assessed overall CS rates. One RCT, implemented in the USA, compared the effects of midwife-led labour and birth care provided in a birth centre with routine labour and birth care provided in a labour ward. This study observed no significant difference in overall CSs between groups (midwife-led: 5/234; routine: 1/253; *p* > 0.05) [39]. The other study, a RCT conducted in China, compared the effects of a midwife-led pregnancy clinic with routine obstetrician-led pregnancy care. This study found that women allocated to the intervention group were less likely to have a CS compared with women in obstetrician-led care group (I: 18/53; C: 30/53; 95% CI for difference − 41.60 to − 3.69) [40].

#### Planned caesarean section

**Fig. 3:**
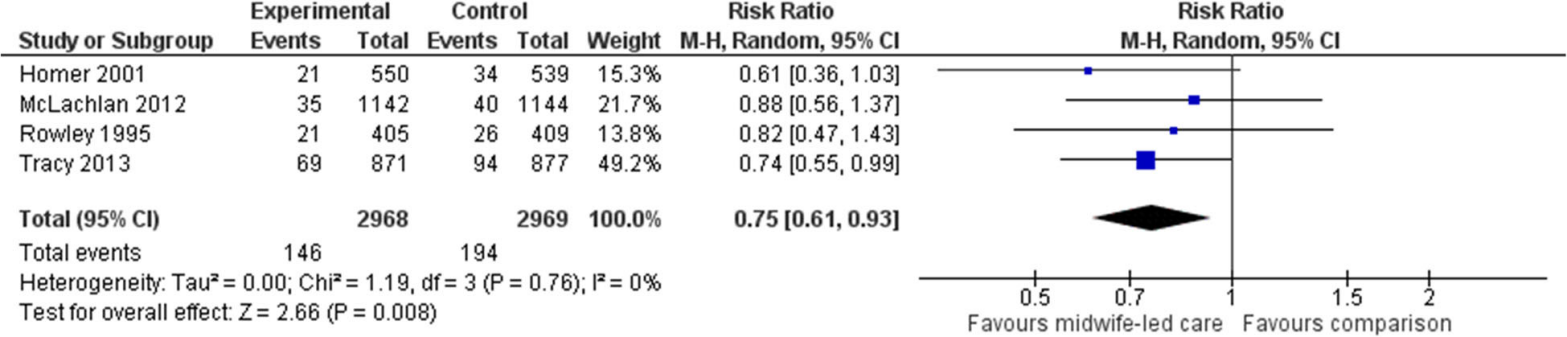
Forest plot for the outcome ‘planned caesarean section’ in the selected RCTs, comparing midwife-led models of care (implemented across pregnancy, labour and birth, and the postnatal period) with standard care

Four studies (*n* = 5937 participants) were included in a meta-analysis comparing midwife-led models of care with a comparator group for planned CSs. Women allocated to midwife-led care were, on average, less likely to experience a planned CS (average RR 0.75, 95% CI 0.61 to 0.93) (Fig. 3). The χ2 test for heterogeneity was not significant, with heterogeneity considered not important (χ2 = 1.19, *p* = 0.76, I^2^ = 0%). No additional studies utilising midwife-led models of care reported on planned CS as an outcome.

#### Unplanned caesarean section

Four studies (*n* = 5937 participants) were included in a meta-analysis comparing midwife-led models of care with a comparator group for unplanned CSs, with results indicating no significant differences between treatment conditions (average RR 0.87, 95% CI 0.73 to 1.03) (Table 2). The χ2 test for heterogeneity was not significant, with heterogeneity considered not important to moderate (χ2 = 4.55, *p* = 0.21, I^2^ = 34%, Tau^2^ = 0.01). No additional studies utilising midwife-led models of care reported on unplanned CS as an outcome.

#### Induction of labour

Five studies (*n* = 5498 participants) were included in a meta-analysis comparing midwife-led models of care with a comparator group for induction of labour, with results indicating no significant differences between treatment conditions (average RR 0.91, 95% CI 0.79 to 1.04) (Table 2). The χ2 test for heterogeneity was not significant, with heterogeneity considered not important to moderate (χ2 = 6.06, *p* = 0.19, I^2^ = 34%, Tau^2^ = 0.01). No additional studies utilising midwife-led models of care reported on induction of labour as an outcome.

#### Epidural use

Six studies (*n* = 7601 participants) were included in a meta-analysis comparing midwife-led models of care with a comparator group for epidural use. Results indicated that the difference between midwife-led care and the comparator condition was not significant, albeit approaching significance (average RR 0.89, 95% CI 0.79 to 1.00, *p* = 0.05) (Table 2). The χ2 test for heterogeneity was significant, with heterogeneity considered moderate to substantial (χ2 = 10.82, *p* = 0.06, I^2^ = 54%, Tau^2^ = 0.01). No additional studies utilising midwife-led models of care reported on epidural use as an outcome.

#### Labour augmentation

The outcome of labour augmentation incorporates the use of intravenous oxytocin and artificial rupture of the membranes (amniotomy) to increase the frequency, duration and intensity of contractions after the onset of spontaneous labour [41]. Five studies (*n* = 5498 participants) were included in a meta-analysis comparing midwife-led models of care with a comparator group for labour augmentation. Results indicated that the difference between midwife-led care and the comparator condition was not significant (average RR 0.97, 95% CI 0.73 to 1.29) (Table 2). The χ2 test for heterogeneity was significant, with heterogeneity considered considerable (χ2 = 48.99, *p* <  0.00001, I^2^ = 92%, Tau^2^ = 0.09). No additional studies utilising midwife-led models of care reported on labour augmentation as an outcome.

#### Instrumental vaginal delivery

The outcome of instrumental vaginal delivery encompasses the utilisation of either forceps or a vacuum device to assist in the vaginal delivery of a foetus [42]. Four studies (*n* = 6776 participants) were included in a meta-analysis comparing midwife-led models of care with a comparator group for instrumental vaginal delivery. Results indicated that the difference between midwife-led care and the comparator condition was not significant (average RR 0.96, 95% CI 0.86 to 1.07) (Table 2). The χ2 test for heterogeneity was not significant, with heterogeneity considered not important (χ2 = 1.82, *p* = 0.61, I^2^ = 0%). No additional studies utilising midwife-led models of care reported on instrumental vaginal delivery as an outcome.

#### Episiotomy

**Fig. 4:**
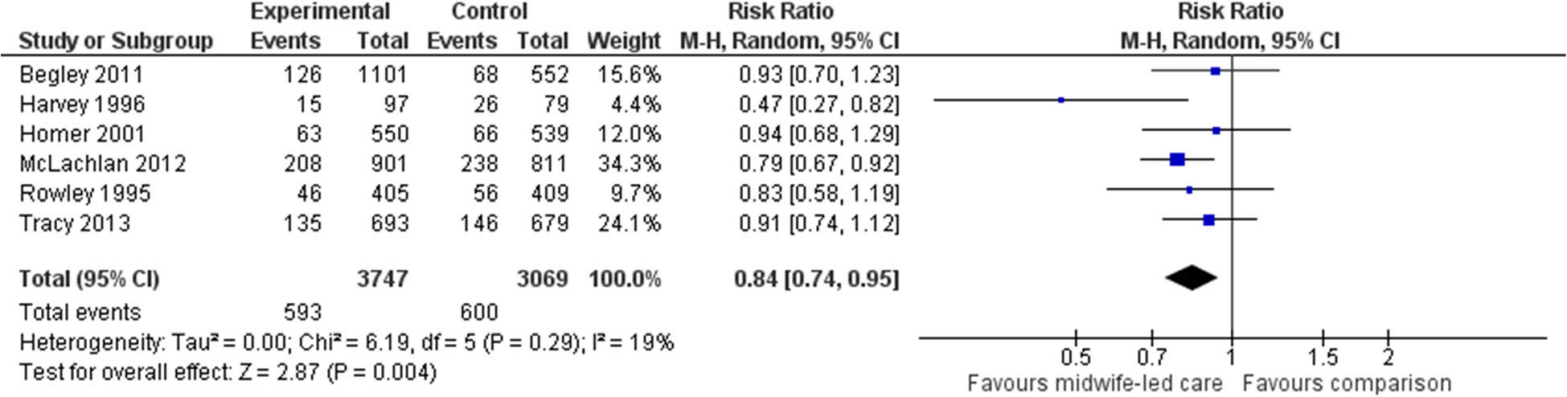
Forest plot for the outcome ‘episiotomy’ in the selected RCTs, comparing midwife-led models of care (implemented across pregnancy, labour and birth, and the postnatal period) with standard care

Six studies (*n* = 6816 participants) were included in a meta-analysis comparing midwife-led models of care with a comparator group for episiotomy. Women allocated to midwife-led care were, on average, less likely to experience an episiotomy (average RR 0.84, 95% CI 0.74 to 0.95) (Fig. 4). The χ2 test for heterogeneity was not significant, with heterogeneity considered not important (χ2 = 6.19, *p* = 0.29, I^2^ = 19%). Narratively, one RCT that compared labour and birth care provided by midwives in a birth centre with labour and birth care provided in a labour ward found that women who were allocated to midwife-led care received significantly fewer episiotomies (I: 24/222; C: 87/246; *p* <  0.0005) [39].

#### Admission during pregnancy

Four studies (*n* = 5304 participants) were included in a meta-analysis comparing midwife-led models of care with a comparator group for admission during pregnancy. Results indicated that the difference between midwife-led care and the comparator condition was not significant (average RR 0.94, 95% CI 0.80 to 1.11) (Table 2). The χ2 test for heterogeneity was significant, with heterogeneity considered moderate to substantial (χ2 = 7.06, *p* = 0.07, I^2^ = 58%, Tau^2^ = 0.02). No additional studies utilising midwife-led models of care reported on admission during pregnancy as an outcome.

#### Apgar scores (< 7 at 5 min)

Three studies (*n* = 4711 participants) were included in a meta-analysis comparing midwife-led models of care with a comparator group for Apgar scores of < 7 at 5 min. Results indicated that the difference between midwife-led care and the comparator condition was not significant (average RR 0.94, 95% CI 0.66 to 1.33) (Table 2). The χ2 test for heterogeneity was not significant, with heterogeneity considered not important (χ2 = 0.88, *p* = 0.64, I^2^ = 0%). Narratively, five additional RCTs utilising midwife-led care reported on Apgar scores as an outcome. These RCTs could not be included in the meta-analysis due to either variations in Apgar scoring criteria (e.g. Apgar cut off < 8), or the implementation of interventions in pregnancy or labour and birth only. However, all five additional RCTs similarly observed no significant differences in Apgar scores between intervention and comparator conditions at 5 min [31–33, 39, 40].

#### Admission to special care unit/neonatal intensive care unit

The meta-analysis for admission to a special care unit (SCU)/neonatal intensive care unit (NICU) incorporated data from five studies (*n* = 6599 participants). Three studies used the combined outcome of SCN/NICU admission [29, 30, 32]; one study used the outcome of NICU admission [34]; and one study used the outcome of SCN admission [31]. Results of the meta-analysis indicated that the difference between midwife-led care and the comparator condition was not significant (average RR 0.80, 95% CI 0.62 to 1.04) (Table 2). The χ2 test for heterogeneity was significant, with heterogeneity considered moderate to substantial (χ2 = 8.54, *p* = 0.07, I^2^ = 53%, Tau^2^ = 0.04).

#### Narrative synthesis of secondary outcomes

A number of additional secondary outcomes of interest could not be included in a meta-analysis of midwife-led models of care. Birthweight and neonatal mortality were assessed within three studies utilising midwife-led care throughout pregnancy, labour and birth, and postnatal periods. Reporting of outcomes varied, preventing conduct of a meta-analysis, however all three studies found no significant differences between midwife-led care and the comparator condition [29, 34]. Postpartum haemorrhage was assessed in two studies (means of measurement not specified), both of which found no significant differences [31, 33]. Two studies reported on postnatal stay (1 day or less; and 0–2 days) as an outcome, with both studies finding that women experiencing midwife-led care were significantly more likely to have shorter postnatal stays [30, 31]. The outcomes of estimated blood loss and perineal trauma were reported within four studies each, however outcome categories varied. Only one of the four studies, an RCT comparing midwife-led labour and birth care in a birthing suite, with labour and birth care in a labour ward, observed fewer 3rd and 4th degree perineal tears among women allocated to midwife-led care [39]. Differences between treatment groups in the four studies reporting on estimated blood loss were predominantly not significant (one blood loss category, < 500 ml, displayed significance in one study [30]). Lastly, in the two studies reporting on continuous electronic foetal monitoring (EFM) as an outcome, one study observed a significant reduction in risk of continuous EFM for women allocated to the midwife-led group [31].

### Continuous midwifery care

#### Overall caesarean section

Three studies (*n* = 7428 participants) were included in a meta-analysis comparing continuous midwifery care during labour and birth with a comparator group for overall CS, including planned and unplanned CSs. Results indicated that the difference between continuous midwifery care and the comparator condition was not significant (average RR 0.85, 95% CI 0.59 to 1.23) (Table 2). The χ2 test for heterogeneity was not significant, with heterogeneity considered moderate to substantial (χ2 = 4.27, *p* = 0.12, I^2^ = 53%, Tau^2^ = 0.06).

#### Narrative synthesis of secondary outcomes

A number of additional secondary outcomes of interest could not be included in a meta-analysis of continuous midwifery care (< 3 studies per outcome per intervention type). Two included studies [35, 36] similarly reported on instrumental vaginal delivery, epidural use, perineal tear, and neonatal admission to higher-level care/NICU, with both studies observing no significant differences between continuous midwifery care and comparator groups. Apgar scores of < 7 at 5 min and birth weight were also reported in two included studies utilising continuous midwifery care [36, 37], with no significant differences found in either study. No differential treatment effects for use of oxytocin in labour were observed within two included studies [35, 37]. Only one RCT utilising continuous midwifery care reported on the outcomes of episiotomy, postpartum haemorrhage, continuous EFM, labour augmentation and neonatal mortality [36]. Significant differences (at the *p* < 0.001 level) were only observed in one outcome; women in the continuous midwifery care group were less likely to have continuous EFM.

### Audit and feedback to promote implementation of evidence-based practice

One included study utilising a cluster RCT design investigated the effects of a multifaceted 1.5-year intervention [27]. The intervention involved audits of CS indications, the provision of formal and informal feedback to health professionals, and implementation of best practices. Small, but significant reductions were observed in CS rate from pre-intervention to post-intervention periods among the intervention group compared with the control (adjusted OR for incremental change over time 0.90, 95% CI 0.80 to 0.99). CS rate was significantly reduced among women with low-risk pregnancies (adj. Risk difference − 1.7, 95% CI − 3.0 to − 0.3), but not among high-risk pregnancies (*p* = 0.35). With regard to secondary outcomes, the intervention group also displayed reductions in major neonatal morbidity (adj. Risk difference, − 0.7, 95% CI − 1.3 to − 0.1), and a smaller increase in minor neonatal morbidity compared to the control (adj. Risk difference − 1.7, 95% CI − 2.6 to − 0.9). No significant differences were observed between groups with regard to changes in minor and major maternal morbidity [27].

### Labour assessment triage

A RCT in Canada that examined the effects of labour assessment and triage at home versus telephone advice found no difference in CS rates between groups of healthy, nulliparous women (RR 1.12, 95% CI 0.94 to 1.32) [38]. Differential treatment effects were not observed for the secondary outcomes of epidural use, labour augmentation, instrumental vaginal delivery, Apgar scores, and neonatal admission to higher-level care units.

### Hospital policy of mandatory second opinion for caesarean section

One included study, a cluster RCT in Latin America, examined the effects of a mandatory second opinion policy for CS [26]. This study observed a small, but significant reduction in CS rates between matched hospitals (mean difference in rate change between matched hospitals: -1.9, 95% CI − 3.8 to − 0.1). Secondary analysis by planned and unplanned CSs revealed a significant difference in unplanned CS rates between matched hospitals (RR -2.2, 95% CI − 4.3 to − 0.1), compared with no difference in planned CS rates (0.2, 95% CI − 1.4 to 1.8). The implementation of the mandatory second opinion policy had no significant impact on the secondary outcomes of instrumental vaginal delivery, neonatal and maternal mortality, neonatal and perinatal mortality, neonatal and maternal admission to intensive care, and women’s satisfaction with the care process [26].

### Hospital protocols for pregnancy complications

One study, utilising a non-concurrent quasi-experimental controlled design, evaluated the effectiveness of protocols for pregnancy complications on CS rate [28]. The study was implemented in Iran and compared CS rates among women with pregnancy complications for periods before and after the initiation of new Ministry of Health and Medical Education protocols. The study found a significant increase in CS rate following the implementation of pregnancy complication protocols (CS rate: intervention phase: 67.8%, control phase 48.8%, *p* = 0.001). With regard to secondary outcomes, duration of hospitalisation, number of specialist visits, and occurrence of complications post-discharge also significantly increased within the intervention phase compared with the control phase (*p* = 0.001) [28].

### Risk of bias/quality appraisal

Twelve RCTs clearly described the utilisation of genuine *random sequence generation* and were categorised as having a low risk of bias (Table 3). Two RCTs did not adequately detail their randomisation process and were classified as unclear [37, 39]. Twelve RCTs were rated low risk of bias for *allocation concealment*, while the remaining two RCTs [27, 34] were judged as unclear due to insufficient information. Classifications for *blinding of participants and personnel* varied between studies; five RCTs were rated as high risk of bias [30, 31, 34–36]; four RCTs as unclear risk of bias [29, 32, 37, 40]; and the remaining five RCTs as low risk of bias [26, 27, 33, 38, 39]. The majority of included studies were judged as low risk of bias for *blinding of outcome assessment* (k = 9). An additional four RCTs were rated as unclear [31, 32, 36, 37], while one RCT was classified as high risk of bias [34]. With regard to *incomplete outcome data*, most RCTs reported loss to follow-up rates of < 20% and utilised an intention-to-treat approach, and were subsequently judged as low risk of bias; one RCT did not provide sufficient information and was rated as unclear [34]. The majority of RCTs (k = 10) were classified as unclear for *selective outcome reporting* because protocols were not available to determine if all outcome data collected were reported. Additionally, no potential *other sources of bias* were identified within most included studies (k = 10).

**Table 3:** Risk of bias summary for studies utilising an RCT/cluster RCT design

Study ID	Random sequence generation *(selection bias)*	Allocation concealment *(selection bias)*	Blinding of participants and personnel *(performance bias)*	Blinding of outcome assessment *(detection bias)*	Incomplete outcome data *(attrition bias)*	Selective reporting *(reporting bias)*	Other sources of bias
Althabe [2004]	+	+	+	+	+	+	?
Begley [2011]	+	+	–	?	+	?	+
Chaillet [2015]	+	?	+	+	+	+	?
Chambliss [1992]	?	+	+	+	+	?	+
Gagnon [1997]	+	+	–	+	+	?	+
Gu [2013]	+	+	?	+	+	?	+
Harvey [1996]	+	+	?	?	+	?	?
Hodnett [2002]	+	+	–	?	+	?	+
Homer [2001]	+	+	+	+	+	+	+
Janssen [2006]	+	+	+	+	+	?	?
Kashanian [2010]	?	+	?	?	+	?	+
McLaughlin [2012]	+	+	?	+	+	?	+
Rowley [1995]	+	?	–	–	?	?	+
Tracy [2013]	+	+	–	+	+	+	+

Note: Low risk of bias (+); High risk of bias (−); Unclear risk of bias (?)

The global rating for the one study utilising a quasi-experimental controlled design [28] was classified as ‘weak’, due to the occurrence of two or more components rated as ‘weak’. Specifically, the *selection bias*, *confounders*, and *blinding* components were rated as ‘weak’; the *study design* and *data collection method* components were rated as ‘moderate’; and the *withdrawals and dropouts* component rated as ‘strong’.

## Discussion

This systematic review including meta-analyses synthesised published evidence and quantified the effects of a subset of maternity service organisational interventions on overall, planned, and unplanned CSs, in addition to a suite of relevant secondary birth outcomes. Results of the separate meta-analyses indicated that women allocated to midwife-led models of care implemented across pregnancy, labour and birth and postnatal periods were, on average, less likely to experience CS (overall), planned CS, and episiotomy compared with women allocated to usual care.

For the meta-analysis of midwife-led models of care, the significant reduction in risk of CS observed in the present review is inconsistent with that observed in a Cochrane review of midwife-led continuity models. No differences between groups were observed in the Cochrane review for overall CS [15]. This discrepancy in effects may largely be attributed to the differences in eligibility criteria between reviews. Unlike the Cochrane review, the present study tightly restricted eligibility to interventions with a primary aim of decreasing CSs. It is therefore not surprising that midwife-led models of care specifically designed to improve rates of physiological birth displayed significant risk reductions in CS rates, when compared to midwife-led models with varied aims. Consistent across both reviews however, was a significant finding for the secondary outcome of episiotomy, with comparable significant risk ratios observed in both reviews (RRs of 0.84). No comparison can be made between reviews for the outcome of planned CS because it was not included as an outcome of interest in the Cochrane review.

The non-significant meta-analysis finding for the effect of continuous midwifery support on overall CS in the present review also differs with that observed in a Cochrane review of continuous one-to-one labour support. The Cochrane review found that women allocated to continuous one-to-one support were less likely to have a CS (low-quality evidence) [16]. Considerable variations in eligibility criteria between reviews may explain this inconsistency. In contrast with the present study, the Cochrane review did not limit inclusion of studies to interventions designed to decease CS rates, and additionally included interventions that utilised informal support persons and doulas within interventions. As a result, the number of studies included in the meta-analysis in the present study was considerably smaller than that of the Cochrane review (k = 3 vs k = 24).

The narrative synthesis of results has additionally identified audit and feedback, and a hospital policy of mandatory second opinion for CS, as interventions that may have potential to reduce CS rates. Both interventions types were utilised within only one included study, with both interventions resulting in small but significant differences favouring the intervention [26, 27]. Audit and feedback is defined in the literature as *‘a summary of the clinical performance of healthcare provider(s) over a specified period of time’* [43]. A previous meta-analysis of evidence-based organisational interventions found audit and feedback as effective in reducing CS rates [14]. However, a number of studies included in the previous meta-analysis were excluded in the present review due to differing classifications in study design, and the requirement for CS to be a primary outcome. With regard to the one study which implemented a hospital policy of mandatory second opinion for CS, secondary analysis revealed the improvement of CS rates were concentrated among unplanned CSs. This finding was not unexpected given the intervention was implemented during labour and birth [26].

A surprising result highlighted within one included study was the significant increase in CS rate observed following the implementation of Iranian Ministry of Health and Medical Education pregnancy complication protocols. The authors of this study acknowledged that the introduction of new protocols may have increased visibility of CS indications among health professionals. However, the authors also acknowledged that the developed protocols were not based on national evidence, but rather extracted from text books, and were therefore unlikely to reflect contemporary recommendations. Additionally, the researchers used a quasi-experimental design with a non-concurrent control group which limits the strength of the findings.

Maternity service organisational interventions are an important consideration given increasing rates of CSs and potential for maternal and perinatal morbidity. As reflected by the publication date of included studies (73% of studies published 2001+), this is an expanding field of research with considerable scope for advancement. To increase the strength of future reviews on the topic, primary studies should strive for consistency in the reporting of outcomes. Studies included in this review varied in the reporting of routinely documented outcomes (e.g. Apgar score cut-offs, maternal blood loss) which subsequently restricted the number of studies included within specific meta-analyses. Future primary studies in the field should also be conducted and reported according to recognised reporting standards, such as the CONSORT statement. Furthermore, as this review has demonstrated, midwife-led models of care are associated with reductions in CS rates. This meta-analysis included interventions utilising caseload midwifery and team midwifery models. More interventions utilising either caseload or team midwifery approaches would enable separate meta-analyses to be performed for each organisational approach, and enable publication bias to be adequately investigated. Standardisation of terminology related to models of care such as midwife-led, caseload and continuity of midwife carer will provide clarification of future analysis and interpretation of results. Moreover, interventions utilising audit and feedback, as well as a hospital policy of mandatory second opinion for CS were identified in the present review as potentially effective for improving CS rates. To enable the quantification of intervention effects, additional primary studies that utilise these approaches alone or in combination are required. It may also be beneficial to study maternity care in OECD countries with low CS rates to identify and study innovations and interventions related to the organisation of care.

The conduct and reporting of this review adhered to the PRISMA statement and utilised systematic and rigorous methods based on Cochrane Collaboration recommendations. The inclusion criteria tightly restricted eligibility to studies that utilised maternity service organisational interventions with a primary outcome of CS. Language or setting restrictions were not applied to the search, which increased the opportunity to include non-English studies. However, no studies published in languages other than English were included in the final set of studies. Had the search strategy intentionally utilised alternative language/country specific databases, the pool of included studies may have been larger, thereby increasing the generalisability of results to additional countries and health settings. An additional consideration that should be noted is the inability to assess publication bias and small study effects due to insufficient numbers of included studies. Planned subgroup analyses, using the Robson classification system, were also not possible due to the included studies being comprised of maternal participant groups that were either not described in sufficient detail, or encompassed a diverse mix of maternal groups (not separated in analysis). This prevented the determination of intervention effects for any of the ten Robson classification maternal groups.

## Conclusions

The findings of this systematic review indicate that women allocated to midwife-led models of care implemented across pregnancy, labour, birth, and the postnatal period were, on average, less likely to experience CS (overall), planned CS, and episiotomy compared with women allocated to routine care. Additionally, the findings suggest audit and feedback, and a hospital policy of mandatory second opinion for CS, are potential interventions that may reduce CS rates. Further research is required to investigate these latter interventions. Given the findings of this review, maternity service leaders should consider the adoption of midwife-led models of care across the maternity episode within their organisations. On the basis that the majority of studies utilising midwife-led models included samples of women classified as being at low-risk for complications (or able to be classified as low-risk), the adoption of midwife-led models of care may be particularly suitable for this maternal group.

## Additional file

Additional file 1: Example search strategy for MEDLINE. (DOCX 16 kb)

## Data Availability

All data generated or analysed during this study are included in this published article and its additional file.

## Abbreviations

CI: Confidence Interval
CS: Caesarean Section
EFM: Electronic Foetal Monitoring
NICU: Neonatal Intensive Care Unit
OECD: Organisation for Economic Cooperation and Development
PRISMA: Preferred Reporting Items for Systematic reviews and Meta-Analyses
RCTs: Randomised Controlled Trials
RR: Risk Ratio
SCU: Special Care Unit
