# Supplementary material for: Maternity service organisational interventions that aim to reduce caesarean section: a systematic review and meta-analyses
Source: BMC Pregnancy Childbirth. 2019 Jul 9;19:206. doi: 10.1186/s12884-019-2351-2 (PMC6615143; doi:10.1186/s12884-019-2351-2)
Supplement: Supplementary file 1 — Example search strategy for MEDLINE. (DOCX 16 kb) [file 12884_2019_2351_MOESM1_ESM.docx]

**Additional file 1: Box 1. Example search strategy for MEDLINE**

1. Cesarean Section/

2. ((caesarean or cesarean) adj (section? or birth? or deliver$)).ti,ab.

3. (c-section? or c section).ti,ab.

4. ((Peri natal or peri-natal) adj care).ti,ab.

5. or/1-4

6. *health maintenance organizations/

7. exp Organizational Innovation/

8. exp Health Services/

9. Health Facilities/

10. 9

11. exp Hospitals/

12. exp Hospital Restructuring/

13. exp Health Planning/

14. Health Policy/

15. Clinical Governance/

16. exp Maternal Health Services/

17. exp "Organization and Administration"/

18. Program Development/

19. Capacity Building/

20. exp "Delivery of Health Care"/

21. "Attitude of Health Personnel"/

22. exp "Quality of Health Care"/

23. exp "Risk Management"/

24. adverse event.ti,ab.

25. exp Patient Care Team/

26. interdisciplinary.ti,ab.

27. exp "Interprofessional Relations"/

28. exp "Quality Assurance, Health Care"/

29. exp Motivation/

30. exp safety/ or patient safety/

31. organizational culture/

32. ((program* or facilit*) adj1 (health or care or intervention*)).ti,ab.

33. capacity building.ti,ab.

34. (organi* adj2 (learning or change or changing or management or restructure or redesign or develop*)).ti,ab.

35. ((quality or safety or change) adj2 (program or improve*)).ti,ab.

36. or/6-35

37. and/5,36

38. exp Morbidity/

39. exp Mortality/

40. exp risk/

41. ae.fs.

42. co.fs.

43. audit*.ti,ab.

44. or/38-43

45. 37 and 44

46. randomized controlled trial.pt.

47. controlled clinical trial.pt.

48. randomized.ab.

49. placebo.ab.

50. drug therapy.fs.

51. randomly.ab.

52. trial.ab.

53. groups.ab.

54. or/46-53

55. exp animals/ not humans.sh.

56. 54 not 55

57. and/45,56

58. limit 57 to yr="1980-2017"
